# Molecular mechanisms of ferroptosis and its antitumor applications in natural products: Ferroptosis and applications in natural products

**DOI:** 10.3724/abbs.2023120

**Published:** 2023-07-05

**Authors:** Dianping Yu, Qun Wang, Qing Zhang, Minchen Cai, Sanhong Liu, Weidong Zhang

**Affiliations:** 1 Shanghai Frontiers Science Center of TCM Chemical Biology Institute of Interdisciplinary Integrative Medicine Research Shanghai University of Traditional Chinese Medicine Shanghai 201203 China; 2 Department of Phytochemistry School of Pharmacy Second Military Medical University Shanghai 200433 China; 3 The Research Center for Traditional Chinese Medicine Shanghai Institute of Infectious Diseases and Biosecurity Shanghai University of Traditional Chinese Medicine Shanghai 201203 China

**Keywords:** ferroptosis, natural product, antitumor, molecular mechanism

## Abstract

Ferroptosis, an iron-dependent form of regulated cell death, results in lipid peroxidation of polyunsaturated fatty acids in the cell membrane, which is catalyzed by iron ions and accumulated to lethal levels. It is mechanistically distinct from other forms of cell death, such as apoptosis, pyroptosis, and necroptosis, so it may address the problem of cancer resistance to apoptosis and provide new therapeutic strategies for cancer treatment, which has been intensively studied over the past few years. Notably, considerable advances have been made in the antitumor research of natural products due to their multitargets and few side effects. According to research, natural products can also induce ferroptosis in cancer therapies. In this review we summarize the molecular mechanisms of ferroptosis, introduce the key regulatory genes of ferroptosis, and discuss the progress of natural product research in the field of ferroptosis to provide theoretical guidance for research on natural product-induced ferroptosis in tumors.

## Introduction

**Figure 1:**
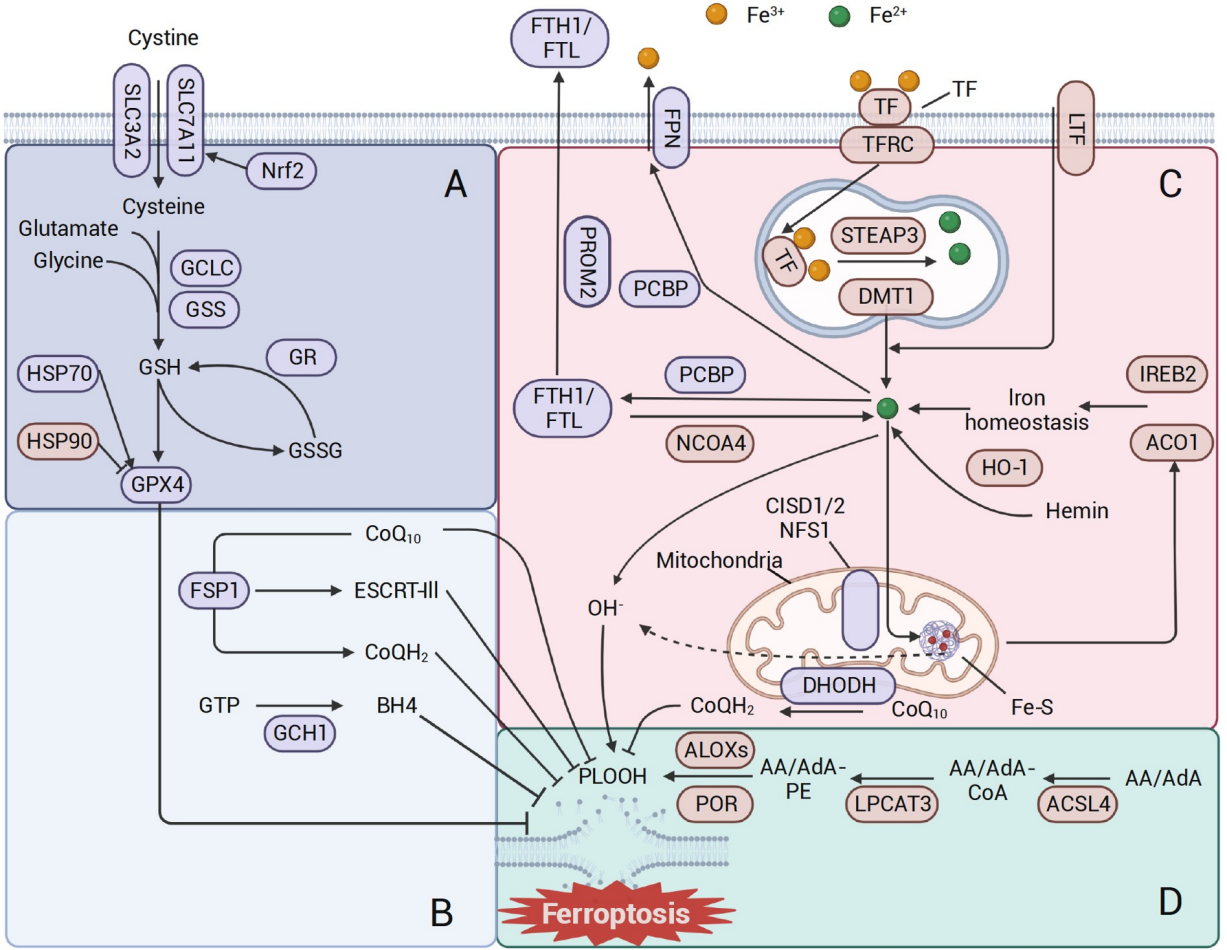
Figure 1 Molecular mechanisms of ferroptosis (A) Canonical ferroptosis antioxidant systems. System xc– delivers cystine into the cell and then catalyzes the synthesis of GSH, thereby maintaining the activity of GPX4 to scavenge lipid peroxidation. (B) Other antioxidant systems. The GCH1-BH4 systems, ESCRT-III membrane repair and FSP1-CoQH2 can also suppress ferroptosis. (C) Iron metabolism. Many proteins regulate iron import, utilization, storage and export to balance iron level in cells. (D) Lipid peroxidation. PUFAs are synthesized in lipid peroxidation by the catalysis of ACSL4, LPCAT3, and ALOX.

Ferroptosis is a novel form of cell death proposed by Professor Stockwell in 2012. When ferrous ions promote lipid peroxidation in a manner similar to the Fenton reaction and accumulate to lethal levels, they rupture the membrane and cause cell death [1]. It is a dynamic process in that cells seek a balance between oxidative systems and ferroptosis defense systems during metabolism. Once the cellular oxidative activity intensifies or the antioxidant capacity is suppressed, ferroptosis occurs [ 2– 6] ( Figure 1). Tumor cells need hypermetabolism and high reactive oxygen level to work, but these are more likely to induce ferroptosis than normal cells. Ferroptosis activates tumor suppressors, stops cancer progression, and establishes a natural barrier for the body [ 7, 8]. However, oncogenic signal-mediated ferroptosis resistance contributes to tumor proliferation, metastasis, and treatment resistance [ 4, 7, 9]. Although tumor cells modulate ferroptosis-associated proteins to compensate for defense systems and survive temporarily, ferroptosis is a targetable vulnerability in cancer therapy. Thus, tumor cells face a dilemma: suppress metabolism and oxidative stress or promote ferroptosis defense mechanisms.

Targeting ferroptosis may present new therapeutic opportunities for those who are drug resistant or insensitive to conventional therapies. Additionally, many studies have suggested that ferroptosis plays an important role in tumor suppression and immunity [ 9 , 10]. The proinflammatory factors produced by ferroptosis induce the tumor immune response and inhibit tumor growth, which solves the problem that apoptosis cannot induce a sufficient immune response because proinflammatory factors are removed rapidly [11]. Nevertheless, the regulatory mechanism of ferroptosis is still unknown in many aspects, as it involves amino acid, lipid metabolism, energy metabolism, redox, and iron homeostasis [10].

This review summarizes the latest views on the mechanisms of ferroptosis regulation, presents the key factors regulating ferroptosis, and discusses the progress in natural product research on ferroptosis.

## Features of Ferroptosis

The morphological features of apoptosis are nuclear fragmentation, cell shrinkage, and apoptotic body formation [12]; necrosis is morphologically characterized by swelling of the cytoplasm and organelles [13]; and autophagy results in the formation of classical autophagic vesicles [13]. In contrast, ferroptosis is mainly characterized by alterations in mitochondria, including reduced volume, increased membrane density, reduced or absence of cristae, and ruptured outer membranes [1]. Biochemically, ferroptosis is characterized by glutathione (GSH) depletion, glutathione peroxidase 4 (GXP4) inactivation, aggregation of iron ions and lipid peroxide aggregation [1].

## Regulatory Mechanisms of Ferroptosis

### Iron metabolism

Ferrous ions play an important role in the ferroptosis regulatory network, mainly through the Fenton reaction and activation of iron-containing enzymes, which produce large amounts of lipid peroxides while supplying energy to cells [ 14, 15]. Thus, iron chelators can significantly inhibit ferroptosis [16]. Iron metabolism includes the uptake, export, storage, and utilization of iron. Cells maintain intracellular iron levels at an appropriate level by regulating iron metabolism. Dietary uptake and recycling of iron from senescent erythrocytes by macrophages are the main sources of iron. Fe ^3+^ binds to serum transferrin (TF) or lactotransferrin (LTF), which is then recognized by the transferrin receptor (TFRC) in the cell membrane [16]. Upon TFRC entering endosomes, Fe ^3+^ is reduced to Fe ^2+^ by six-transmembrane epithelial antigen of prostate 3 (STEAP3); it further passes through solute carrier family 11 member 2 (SLC11A2/DMT1) and is released into the cytoplasmic labile iron pool (LIP) [17]. Poly(C)-binding protein family proteins (PCBP) are ferrous ion chaperone proteins that bind with free Fe ^2+^ in the cytoplasm and deliver it to the corresponding proteins involved in iron utilization, storage, and export. First, Fe ^2+^ is transferred into mitochondria by mitochondrial transport proteins (SLC25A28 and SLC25A37) and then participates in various aspects of metabolic and biochemical processes, including energy metabolism, the synthesis of hemoglobin and iron-sulfur proteins, and storage in mitochondrial ferritin [18]. Normally, cancer cells utilize iron through the cysteine desulfurase (NFS1)-iron-sulfur cluster assembly enzyme (ISCU)-CDGSH iron-sulfur domain-containing protein 1 and 2 (CISD1/2) axis to inhibit mitochondrial lipid peroxidation and ferroptosis; when Fe ^2+^ in mitochondria is overloaded, it induces enzyme inactivation, impaired iron metabolism and ferroptosis [ 19– 21 ]. Second, PCBP transfers Fe ^2+^ to ferritin (FTH1/FTL) stores or exports excess Fe ^2+^ out of cells via the transporter SLC40A1 (FPN). In addition, prominin 2 (PROM2), a pentaspan transmembrane glycoprotein, exports ferritin and its storage iron through exosomes [22]. Theoretically, each step of iron metabolism provides the possibility for drugs to induce ferroptosis and combat tumors, and extensive evidence suggests that ferroptosis induced by iron overload inhibits tumor cell growth and proliferation [ 23, 24]. For instance, β-elemene and cetuximab upregulate heme oxygenase 1 (HO-1) and transferrin to induce ferroptosis in KRAS mutant colorectal cancer [25]. Temozolomide induces ferroptosis in glioblastoma cells via DMT1 [26]. Silencing of *PCBP1* mediates ferroptosis in head and neck cancer [27].

Abnormalities in genes related to iron metabolism usually induce ferroptosis in tumor cells. Iron responsive element binding protein 2 (IREB2) is one of the major regulators of iron metabolism and regulates ferroptosis by affecting the expressions of TFRC, ISCU, FTH1, and FTL [ 1, 28]. Erastin induces ferroptosis in fibrosarcoma and breast cancer by regulating ferrous ion levels via IREB2 [ 1, 29]. Nuclear receptor coactivator 4 (NCOA4)-mediated ferritinophagy recruits FTH to lysosomes for degradation, generating large amounts of ferrous ions in the labile iron pool [30]. Therefore, activation of NCOA4 and lysosomal activity elevates the level of ferrous ions and promotes ferroptosis [ 31, 32]. Additionally, inhibition of cytosolic glutamate oxaloacetate transaminase 1 (GOT1) enhances ferritinophagy and promotes ferroptosis [33]. In addition, a study showed that mitochondrial ferritin (FTMT) inhibits erastin-induced ferroptosis in neuroblastoma cells, suggesting that iron storage proteins play an important role in inhibiting ferroptosis [34]. Similarly, Nrf2 regulates iron metabolism through HO-1, and excessive activation of HO-1 catalyzes heme degradation to Fe ^2+^, which causes noncanonical ferroptosis. However, moderate upregulation of HO-1 may promote its cytoprotective effects by enhancing antioxidant activity [ 35, 36]. Therefore, targeting iron metabolism-related proteins is a potential strategy to induce ferroptosis in tumor cells.

### Lipid peroxidation

#### Nonenzymatic lipid peroxidation

Nonenzymatic lipid peroxidation is a series of chain reactions driven by intracellular radicals, in which Fe ^2+^ and hydrogen peroxide (H _2_O _2_) produce hydroxyl radicals via the Fenton reaction. Hydroxyl radicals extract hydrogen from the PUFA at the phospholipid (PL) sn2 site to form a carbon-centered lipid radical (L ^–^) and subsequently react with molecular oxygen (O _2_) to generate a lipid peroxy radical (LOO ^–^). LOO ^–^ can directly or indirectly form lipid peroxide (LOOH) and a new lipid radical by reacting with the adjacent PUFA or Fe ^2+^ to trigger another chain reaction. If PLOOH cannot be neutralized, the iron-catalyzed amplification reaction leads to an increase in membrane permeability and loss of membrane integrity, and then ferroptosis occurs in the cell. Artemisinin increases cellular free iron and lipid peroxidation and sensitizes cancer cells to ferroptosis [37]. Thus, iron chelators and lipophilic radical scavengers (RTAs) can be effective in preventing ferroptosis [ 10, 38 ].

#### Enzymatic lipid peroxidation

In enzymatic lipid peroxidation, acyl-CoA synthetase long-chain family member 4 (ACSL4) and lysophosphatidylcholine acyltransferase 3 (LPCAT3) are important driving factors. Long-chain PUFAs, mainly free arachidonic acid (AA) or adrenaline (AdA), are bound to CoA by ACSL4 to form the derivatives AA-CoA and AdA-CoA, which are then further processed by LPCAT3 to membrane phosphatidylethanolamine (AA-PE or AdA-PE). Previous studies have shown that the deletion of ACSL4 inhibits ferroptosis in many tumor cells [39]. The peroxidation of PLs, but not FAs, is more significant for ferroptosis; therefore, ACSL4 is thought to be central to ferroptosis [ 40, 41]. Arsenic trioxide induces antitumor ferroptosis by targeting ACSL4 [42]. In contrast, commonly used ALOX inhibitors have been reported to have RTA activity, which challenges the critical role of ALOX in ferroptosis [43]. Shah *et al*. [43] suggested that the importance of the enzymatic lipid peroxidation response for ferroptosis lies in bringing cells to the critical PLOOH threshold, rather than nonenzymatic lipid peroxidation being the actual driver of ferroptosis [44].

### Antioxidant systems

Scavenging of reactive oxygen species (ROS) and lipid peroxidation via the system x _c_^−^-glutathione (GSH)-glutathione peroxidase 4 (GPX4) axis has long been considered a central component of the ferroptosis defense mechanism, in which many classical ferroptosis inducers and inhibitors have been identified ( Figure 1A and Table 1). An increasing number of pathways (mTOR, Nrf2, p53, *etc*.) with proteins (Hsp90 and Hsp70) have been reported for the regulation of system x _c_^−^ and GPX4, enriching the regulatory network of this pathway in ferroptosis [ 7, 45– 47].

**Table:** **Table 1** Regulators of ferroptosis

Regulator	Impact on ferroptosis	Reagent	Ref.
Iron metabolism			
IREB	Regulate the target genes that affect iron homeostasis to balance iron levels	shRNA	[1]
HO-1	Catalyze degradation of heme to iron	Withaferin A BAY 11–7085	[ 35, 55]
HSPB1	Decrease iron level to inhibit erastin-induced ferroptosis	shRNA	[56]
NCOA4	Cargo receptor for ferritinophagy, promote degradation of ferritin to iron	shRNA	[32]
FTH1	Store labile iron	shRNA	[57]
STEAP3	Regulated by FANCD2, convert iron from Fe ^3+^ to Fe ^2+^		[58]
NFS1	Synthesize iron-sulfur clusters using labile iron	shRNA	[59]
Lipid metabolism			
ACSL4	Catalyze PUFA to phospholipids to promote lipid peroxidation	Thiazolidinedione	[ 39, 41]
LPCAT3	Insert acyl groups into lysophospholipids	shRNA	[41]
ALOX	Catalyze the conversion of PUFA into lipid peroxidation	AA861 PD146176	[60]
SQS	Synthesize farnesyl pyrophosphate into squalene, resulting in reduced CoQ10 and failure to scavenge lipid peroxidation	FIN56	[61]
HMGCR	Synthesize mevalonate, the source of CoQ10	Statins	[61]
Antioxidant system			
GPX4	Scavenge lipid peroxidation	RSL-3 FIN56 ML162 FINO2	[ 61– 64]
HO-1	Knockdown enhances erastin-induced ferroptosis	shRNA	[ 57, 65]
Nrf2	Regulate the expression of the antioxidant gene including system x _c_^–^ and FTH1	Trigonelline	[66]
SLC7A11	Import cystine to synthesize GSH	Erastin glutamate Sulfasalazine	[1]
GCLC	Synthesize GSH	BSO	[62]
FSP1	Reduce CoQ10 to CoQH2 to scavenge lipid peroxidation	iFSP1	[49]
GCH1	Synthesize BH4 to scavenge lipid peroxidation	Plasmids	[54]
DHODH	Synthesize CoQH2 to scavenge lipid peroxidation	Brequinar	[50]

Several mechanisms independent of GPX4, such as FSP1, CoQ10, BH4, and the ESCRT-III membrane repair system, have also been reported to prevent ferroptosis and lipid peroxidation ( Figure 1). Ferroptosis suppressor protein 1 (FSP1) uses NAD(P)H to reduce CoQ10 to CoQH2, thereby scavenging lipid peroxidation radicals [ 48, 49]. Unlike FSP1, although dihydrogen phosphate dehydrogenase (DHODH) also neutralizes lipid peroxidation by increasing CoQH2 synthesis, this process occurs mainly in mitochondria, so GPX4 and DHODH can collaborate to enhance the inhibition of mitochondrial lipid peroxidation, but cytoplasmic GPX4 and FSP1 cannot [ 50, 51]. Similarly, GTP cyclohydrolase 1 (GCH1) acts as a radical-trapping antioxidant by generating BH4 to inhibit ferroptosis [ 52– 54].

## Regulators of Ferroptosis

### GPX4

There is no doubt that GPX4 has been the focus of research on intracellular antioxidant mechanisms since the discovery of ferroptosis. Unlike most enzyme families, GPX4 is the only enzyme that can scavenge lipid peroxides in the GPX enzyme family [9]. Similarly, GPX4 is the main enzyme that catalyzes the reduction of PLOOH in cells [67]. It catalyzes phospholipid hydroperoxides (AA/AdA-PE-OOH) into the corresponding phosphatidyl alcohols (PLOH). In addition, GPX4 is a selenoprotein, and selenium increases GPX4 activity via a selenocysteine residue at U46 [ 15, 62, 68 , 69]. Deficiency of GPX4 or inhibition of GPX4 activity by binding to the active site leads to the accumulation of lipid peroxidation, which induces ferroptosis in cells or tissues, and lipophilic radical scavengers inhibit these processes [ 63, 70– 72]. Thus, GPX4 is considered an important inhibitor of ferroptosis, and ample compounds such as gallic acid, withaferin A, and oridonin were reported to induce ferroptosis in tumor cells through downregulation of GPX4 expression.

### System x _c_^–^

Glutathione is a cofactor for many antioxidant enzymes and is also responsible for maintaining the activity of GPX4 [73]. System x _c_^–^ is formed by the polymerization of two core proteins, SLC7A11 (xCT) and SLC3A2 (4F2hc), the former being responsible for the import of extracellular cystine, while the latter displaces intracellular glutamate [70]. Erastin induces ferroptosis by inactivating GPX4 indirectly through the depletion of GSH [1]. A number of compounds have been used to target the system x _c_^–^-GSH axis to promote ferroptosis or enhance sensitivity to ferroptosis and have received FDA approval, such as sulfasalazine and sorafenib [74]. Furthermore, erianin induces ferroptosis in a variety of tumor cells, including lung cancer, renal cell carcinoma, and bladder cancer cells, by inhibiting system xc ^–^ and depleting GSH [ 75– 77]. In conclusion, the system x _c_^–^-GSH pathway is one of the critical upstream mechanisms for the induction of ferroptosis.

### FSP1

The ability of some tumor cells to proliferate without GPX4 has triggered the exploration of pathways other than GPX4 to prevent ferroptosis. Overexpression of FSP1 inhibits ferroptosis and is independent of ACSL4 and PUFA levels [48]. It has been shown that iFSP1, an inhibitor of FSP1, promotes ferroptosis in GPX4-deficient tumor cells [49]. In addition, FSP1 inhibits ferroptosis by activating ESCRT-III-dependent membrane repair [16].

### ALOX

Free and esterified polyunsaturated fatty acids, mainly linoleic acid (LA) and arachidonic acid (AA), are catalyzed by lipoxygenase (ALOX) to produce various lipid hydroperoxides [78]. Studies have shown that inhibition or knockdown of lipoxygenase can inhibit ferroptosis in some cell types [ 79, 60 ]. Similarly, phosphatidylethanolamine binding protein 1 (PEBP1) promotes ferroptosis by directing ALOX15 to recognize polyunsaturated fatty acids on the cell membrane [80]. Although ALOX can sensitize cells to ferroptosis, it is not essential because of other enzymatic or nonenzymatic mechanisms of PL peroxidation. Previous studies suggested that ALOX plays a limited role in ferroptosis because the expression of ALOX is low in cell lines that are commonly studied for ferroptosis, and ALOX inhibitors usually have RTA activity [ 43, 81]. Furthermore, inhibition of ALOX does not prevent ferroptosis in cells lacking GPX4 [82]. Therefore, the regulation of ferroptosis by ALOX still needs further in-depth study.

### ACSL4

ACSL4 is an enzyme involved in fatty acid metabolism that promotes ferroptosis by increasing the PUFA content in phospholipids and is therefore considered a specific biomarker and driver of ferroptosis [74]. It was found that protein kinase C betaII (PKCβII) is first activated and then phosphorylates ACSL4, promoting ACSL4-mediated PUFA-PL synthesis and ultimately leading to positive feedback amplification of ferroptosis [83]. Overexpression of ACSL4 may promote ferroptosis; conversely, deletion of ACSL4 moderates ferroptosis, suggesting that ACSL4 may be one of the main mechanisms of ferroptosis [ 2, 84 – 86].

### Nrf2

Nrf2 is a major transcription factor of oxidative stress signaling that activates a large number of cytoprotective genes involved in multiple aspects of ferroptosis regulation, such as iron metabolism, oxidative defense, and redox systems. Nrf2 can reverse sorafenib-induced ferroptosis and has therefore been identified as an important defense mechanism for ferroptosis [57]. In recent years, a large number of Nrf2 target genes have been identified, covering iron metabolism (SLC40A1, FTH1, HO-1, *etc*.), GSH metabolism (SLC7A11, GPX4, GCLM, *etc*.), and ROS detoxification enzymes (TXNRD1, AKR1C family, NQO1, *etc*.) [74], further suggesting that Nrf2 is a valuable protein in the regulation of ferroptosis. Xiang *et al*. [76] found that inactivation of Nrf2 promoted erianin-induced ferroptosis in bladder cancer. Notably, Nrf2 may play a dual role in tumor progression, with both deficient and high expression of Nrf2 promoting tumor proliferation. Both nobiletin and tagitinin C elevates cellular ferrous ion levels via the Nrf2-HO-1 axis, which promotes ferroptosis in tumor cells [ 87 , 88].

## Natural Products and Their Application for Ferroptosis

The role of natural products in cancer treatment can be summarized as follows: (1) natural products can be used for tumors that are sensitive to ferroptosis [9]; (2) natural products can induce ferroptosis to reverse drug resistance and enhance immunotherapy [ 11, 89]; (3) due to their multitarget characteristics and few side effects, they may induce ferroptosis in tumor cells while exerting protective effects or less toxicity in normal cells [ 90, 91].

### Ferroptosis and natural products in drug resistance

Drug resistance has been a major challenge in the clinical treatment of cancers, and numerous studies have attempted to overcome it. Recent findings suggest that ferroptosis is associated with drug resistance. Using natural compounds to trigger ferroptosis offers great potential for drug-resistant cancers to enhance chemotherapeutic efficacy [89]. Mechanistically, mounting evidence suggests that SLC7A11 is overexpressed in many cancers; in particular, multiple factors reverse tumor suppression by stabilizing or upregulating SLC7A11 in sorafenib- and cisplatin-resistant cells [ 24, 89, 92, 93 ]. Similarly, Nrf2 has been found to be heavily upregulated in drug-resistant cells, since *Nrf2* encodes a large number of antioxidant system proteins [ 24, 36 ]. In addition, suppression of GPX4 is an important strategy to overcome the resistance of cancer to chemotherapy [94]. Therefore, targeting ferroptosis has emerged as a potential therapy for drug resistance. In fact, many studies have reported that natural products induce ferroptosis to overcome drug resistance. Curcumin analog reverses temozolomide resistance in glioblastoma by downregulating GPX4 [95]. Compound 23 isolated from *Jungermannia tetragona* overcomes cisplatin resistance by targeting Prdx I/II and depleting GSH [96]. Furthermore, dihydroartemisinin increases cellular LIP and addresses cisplatin resistance in pancreatic ductal adenocarcinoma [97]. Additionally, artesunate induces ferroptosis in renal cell carcinoma by inhibiting GPX4, which reverses sunitinib resistance [98]. Additionally, natural products such as soyauxinium chloride, epunctanone, and ungeremine were reported to display cytotoxicity toward drug-resistant tumor cells via ferroptosis [ 99– 101].

### Ferroptosis and natural products in immunotherapy

Tumor immunotherapy regulates the immune response and inhibits tumor growth by activating the body’s immune defense system and is considered an important therapy for tumors [ 102, 103 ]. However, poor immunogenicity, immune checkpoints, and immunosuppressive factors enable tumors to establish their unique immunosuppressive microenvironment, which limits the activity of effective T cells (Teffs) and resists the immune system from recognizing and attacking tumors [ 104 – 107]. Therefore, the indications for tumor immunotherapy are limited. First, the lack of tumor-specific antigens in cold (poorly immunogenic) tumors results in the inability of T cells to recognize tumor cells [ 108, 109]. Second, although Teffs bind to tumor cells, tumor cells can avoid attack via immune checkpoints (ICTs), such as PD-L1 and CTLA4 [ 110– 112]. In addition, tumor cells secrete specific factors to induce mast cells, which induce immunosuppression and promote tumor growth [ 113– 115]. Therefore, effective strategies to expand their indications and improve their efficiency have become the key element for cancer immunotherapy research [ 11, 112].

Natural products reshape the immunosuppressive tumor microenvironment and show great potential in enhancing the therapeutic efficacy of cancer immunotherapy. For example, capsaicin, ginsenoside Rg3, and resveratrol induce DAMP exposure to enhance the immunogenic cell death (ICD) effect [ 116– 118]. As adjuvants for vaccines, numerous reports have shown that saponins, polysaccharides and flavonoids from natural products can effectively enhance immunostimulatory effects and reverse immunosuppression [ 119– 123 ]. In addition, berberine, baicalin, and cordycepin downregulate PD-L1 expression in tumor cells [ 124– 126 ], and andrographolide, diosgenin, and geranium promote the efficiency of anti-PD-1/PD-L1 antibodies [ 127, 128 ].

As a novel mode of cell death mentioned in recent years, the relationship between ferroptosis and tumor immunity has attracted the attention of researchers. It was found that cancer cells undergoing ferroptosis could activate the tumor microenvironment through the release of DAMPs and create positive feedback of the immune response [ 129– 131 ]. Similarly, tumor-bearing mice treated with an anti-PD-L1 antibody show ferroptosis characteristics, such as an increase in lipid peroxidation [132]. The lipid peroxidation produced by ferroptosis could serve as a signal that promotes dendritic cell recognition of tumor antigens and improves tumor immunotherapy. After the combination of immunotherapy and ferroptosis inducers, infiltration of cytotoxic T lymphocytes was significantly increased [133]. In addition, immunotherapy may increase sensitivity to ferroptosis; thus, coadministration of ferroptosis inducers may generate a strong immune response and promote ferroptosis against tumors [131]. Lou *et al*. [134] showed that fascaplysin induced ferroptosis while upregulating PD-L1 expression, and increased sensitivity to anti-PD-L1 immunotherapy in non-small cell lung cancer. Although there are fewer reports that natural products activate tumor immune responses via ferroptosis, it is foreseeable that this will be a potential strategy for tumor suppression. Similarly, the cytotoxicity of ferroptosis on immunecells should be noted.

### Advances in natural products in the field of ferroptosis

#### Artemisinin and its derivatives

**Figure 2:**
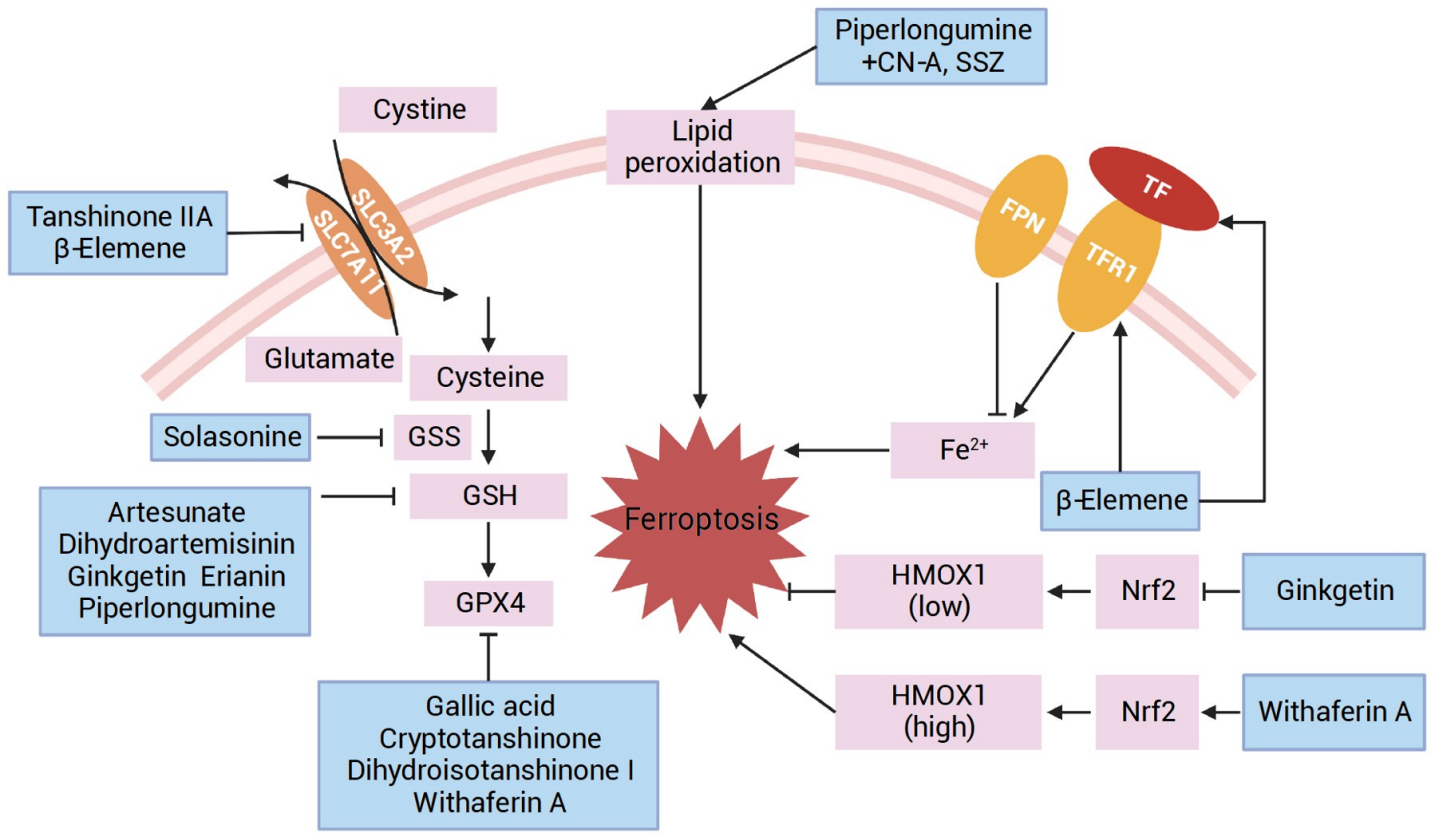
Figure 2 Functions of natural products in inducing ferroptosis

In addition to their antimalarial effects, the antitumor effects of artemisinin and its derivatives have been extensively studied. Artemisinin, dihydroartemisinin, and artesunate, on the one hand, increase ferrous ion level through lysosomal degradation of ferritin or upregulation of NCOA4 and DMT1 levels; on the other hand, they induce ferroptosis through downregulation of GSH and GPX4 levels [ 37, 135, 136] ( Figure 2). According to previous research, artemisinin and its derivatives regulate 20 genes related to iron metabolism, including *SLC40A1*, *IREB*, *FTMT*, and *ISCU*, to induce ROS production [137]. In addition, inhibition of the Nrf2-ARE pathway allows artesunate to reverse cisplatin resistance through ferroptosis, and coadministration of artesunate enhances the sensitivity of hepatocellular carcinoma cells to sorafenib [66].

#### Salvia officinalis extract

Tanshinone extracts include tanshinone IIA, dihydrotanshinone I, and cryptotanshinone. In normal cells, tanshinone IIA inhibits hepatocyte ferroptosis in an atherosclerosis model by upregulating the expressions of GPX4, SLC7A11, and FTH1 [138] ( Figure 2); in hippocampal cells, it inhibits ferroptosis by upregulating HO-1 expression to lower LPO and Fe ^2+^ levels [139]. In contrast, in gastric cancer cells, tanshinone IIA mediates the decrease in GSH and increase in ROS triggered by the downregulation of SLC7A11 level [91]. In addition, cryptotanshinone induces ferroptosis in lung cancer cells through HSPB1 and GPX4 upregulation and IREB2 downregulation [140]. Dihydroisotanshinone I reduces GPX activity or inhibits GPX4 protein expression, increasing malondialdehyde (MDA) and lipid ROS levels [ 141, 142].

#### Piperlongumine

Ferroptosis induced by piperlongumine mainly depends on the inactivation of antioxidant mechanisms and the accumulation of ROS in cells ( Figure 2). In a variety of cancer cell lines, including gastric, pancreatic, and colorectal cancers, piperlongumine inhibits GPX4 activity mainly by reducing GSH, leading to ROS accumulation [143]. It also significantly enhances the anticancer effect of ferroptosis by combining sulfasalazine, oxaliplatin, and APR-246 [144]. In contrast to cancer cells, activation of HO-1 by piperlongumine inhibits ferroptosis in normal cells; *in vivo* studies also showed that piperlongumine attenuates weight loss induced by oxaliplatin treatment [ 145 , 146].

#### Others

In addition to the natural products mentioned above, a large number of compounds have been reported in antitumor studies via ferroptosis mechanisms, encompassing the modulation of iron metabolism, lipid peroxidation, and antioxidant systems. Hence, the main mechanism by which other natural products induce ferroptosis is summarized in Figure 2 and Table 2.

**Table:** **Table 2** Ferroptosis-induced natural products

Compound	Effect	Cancer type	Ref.
Erianin	Downregulate GPX4, GSH, and SLC7A11, and activation of CaM caused an increase in Fe ^2+^	Lung cancer	[77]
Oridonin	Decrease GGT1 activity to block GSH formation and reduce GPX4 activity	Esophageal cancer	[147]
Solasonine	Suppress GPX4 and GSS to increase lipid ROS	Hepatocellular carcinoma	[148]
Pseudolaric acid B	Upregulate p53 to inhibit SLC7A11 and GSH, upregulation of TFRC	Glioma	[149]
Withaferin A	Increase Fe ^2+^ by tragting Keap1 to activate HO-1; inactivates glutathione peroxidase 4	Neuroblastoma	[35]
Actinidia chinensis planch	Suppress GPX4 and SLC7A11 to accumulate ROS	Gastric cancer	[150]
Gambogic acid	Upregulate p53 to inhibit SLC7A11, GSH and GPX4; reduced SOD activity	Melanoma	[151]
Ginkgetin	Decrease expression of SLC7A11 and GPX4, inactivation of Nrf2/HO-1	Non-small cell lung cancer	[152]
Glycyrrhetinic acid	Activate NADPH oxidases, and decrease GSH and GPX4 activity	Triple-negative breast cancer	[152]
Cucurbitacin B	Accumulate iron ions, downregulate the expression of GPX4	Nasopharyngeal cancer	[153]
6-Gingerol	Upregulate LC3-II and NCOA4 to induce ferritinophagy and downregulation of GPX expression	Lung cancer	[154]
Polyphyllin II	Activate NCOA4-induced ferritinophagy to increase iron ion	Hepatocellular carcinoma; KRAS mutation harboring cancer	[ 155, 156 ]
β-Elemene	Upregulate the expression of HO-1 and TF to elevate Fe ^2+^, and downregulation of GPX4, SLC7A11 and GS	KRAS mutant colorectal cancer	[25]
Gallic acid	Promote TFRC, ATF4 and iron ion levels and inhibits GPX4 and SCL7A11	Colorectal cancer	[157]
Oleanicacid	Promote ACSL4 expression and iron ion to accumulate ROS and MDA	Cervical cancer	[158]

## Conclusions and Perspectives

Targeting ferroptosis to antitumor effects will be a hot topic of scientific research, although the mechanism is not fully elucidated and needs further study. However, it is certainly the result of an imbalance between the intracellular redox protective system and iron metabolism. The sensitivity to ferroptosis varies among tumors, and therefore, an in-depth investigation of its different regulatory mechanisms would be instructive for the clinical application of targeting ferroptosis in tumors. Currently, the key regulator GPX4 has become an important therapeutic target for the development of anticancer drugs; as more research is conducted, more factors involved in ferroptosis will be identified, which will provide more targets and treatment options for clinical application.

At the same time, ferroptosis is a double-edged sword; on one hand, we use it to eliminate tumor cells, but on the other hand, we should also be concerned about the damage that ferroptosis may cause to normal cells. For example, GPX4 inhibits TMEM16A-mediated hepatic ischemia/reperfusion injury [159]. Additionally, DHODH inhibits ferroptosis in spinal cord injury [51]; similarly, ferroptosis exacerbates most cardiovascular diseases [160]. Notably, trastuzumab induces severe cardiotoxicity while treating breast cancer via ferroptotic cell death; however, SGLT2 inhibitors eliminate cardiotoxicity and show potent antitumor activity [161]. Targets such as SGLT2 deserve our attention because they can protect normal cells and kill tumor cells simultaneously.

Common clinical drugs have disadvantages such as poor selectivity and toxic side effects, which severely limit their efficacy. Natural products have multipathway and multitarget anticancer properties. How natural products regulate and interfere with ferroptosis for cancer treatment still needs further in-depth research. For example, as mentioned above, tanshinone IIA and piperlongumine can induce ferroptosis in tumor cells while also promoting antioxidation and inhibiting ferroptosis in normal cells [ 90, 145, 146]. Is there a threshold of intracellular Fe ^2+^ and lipid peroxidation levels that would allow tumor cells to undergo ferroptosis while leaving normal cells unaffected? Or whether there is a new target that induces ferroptosis in tumor cells only, once the mechanism is clarified, this will provide great diagnostic and therapeutic value to the clinic. In addition, the link between ferroptosis and other cell death modalities remains to be explored, and if the intrinsic link is clarified, this could provide a theoretical basis for the clinical combination of drugs. As the mechanism of natural products against tumors through ferroptosis will be further investigated, it may lead to new strategies for ferroptosis-based cancer therapy.
